# Healthy lives, enriched minds: the role of physical health and mental health on educational attainment in Northern Ireland

**DOI:** 10.1186/s12889-025-23780-3

**Published:** 2025-08-22

**Authors:** Erin Early, Sarah Miller, Laura Dunne, Dermot O’Reilly

**Affiliations:** 1 School of Applied Social and Policy Sciences, Ulster University, 2-24 York Street, Belfast, BT15 1ED UK; 2 School of Social Sciences, Education and Social Work, Queen’s University Belfast, 69-71 University Street, Belfast, BT7 1HL UK; 3 Administrative Data Research Centre Northern Ireland (ADRC NI), Belfast, UK

**Keywords:** Physical health, Mental health, Attainment, Administrative data, Data linkage

## Abstract

**Background:**

Health is a multifaceted concept with existing evidence in the UK indicating a negative association between educational outcomes and markers of ill-health. Evidence that disaggregates the influence of physical and mental health conditions on educational attainment, using population wide linked administrative data, varies between UK jurisdictions, with a lack of such research evident in Northern Ireland (NI). This study aims to address this gap by investigating the impact of students’ physical and mental health on post-primary attainment in NI. This is a pertinent area as the recent Children and Young People’s Emotional Health and Wellbeing in Education Framework outlined the need for cross-departmental collaboration between education and health.

**Methods:**

Using administrative data that linked the household Census (2011), School Leavers Survey (2010–2014) and School Census (2010–2014), this study examines the associations between young people’s GCSE attainment, physical health and mental health (*n* = 61,373). Multilevel models were executed to account for the nested data structure and wider demographic profile of young people. Interaction terms were also tested between factors such as sex, socio-economic background, and physical and mental ill-health markers to determine their effects on attainment.

**Results:**

The study confirmed the negative associations between lower post-primary attainment and ‘very bad’ self-reported health status (*d =* -1.01, 95% CI: -1.26, -0.75), the presence of a physical health condition (*d =* -0.39, 95% CI: -0.41, -0.36), mental health condition (*d =* -0.91, 95% CI: -1.00, -0.82) and an illness/disability that limited daily activity a little (*d =* -0.48, 95% CI: -0.53, -0.44) or a lot (*d =* -0.98, 95% CI: -1.04, -0.92). Interaction terms were tested, and significant associations were evident between sex and physical health condition, sex and mental health condition, free school meal eligibility and physical health condition, and the presence of both a physical health condition and mental health condition.

**Conclusion:**

This study is the first instance where population wide, linked administrative data is used to examine the associations between educational attainment and students’ physical and mental health in NI. The importance of mental health and the greater educational disadvantage some social groups may experience are key implications for policy.

**Supplementary Information:**

The online version contains supplementary material available at 10.1186/s12889-025-23780-3.

## Background

In recent years, the World Health Organisation (WHO) has reported an increased prevalence of health conditions amongst young people [1]. This is of concern for education research as students’ physical and mental health has been found to be a significant predictor of academic attainment and education outcomes [2, 3]. Evidence indicates that students experiencing physical health conditions or physical ill-health are at risk of underperforming in assessments [4, 5], however this relationship is not clear cut, as some studies report that students with specific chronic physical illnesses have similar or better academic outcomes than those with no chronic illnesses [6]. Mental health is also associated with education outcomes, with evidence highlighting the lower attainment of students with mental health conditions and illnesses [7–10]. However, there is also variation in these trends, depending on the mental health condition in question [8] and existing studies across the international context are limited in their generalisability based on the data quality, sample and explanatory factors used in the analysis [11]. When considering the United Kingdom (UK) context, it is argued that the evidence base for the association between mental health and educational outcomes is less established than elsewhere [11], and this is particularly the case for Northern Ireland because of, but not limited to, the lack of access to linked, high quality administrative data. Across the UK, there are differences in how population wide measures of mental health are captured. For example, the household Census in Scotland and Northern Ireland provide respondents with an option to disclose the presence of a mental health condition, however such option is not available in the Census for England and Wales.

Mental health and physical health are often discussed separately, despite their interlinking relationships that can enable optimum health maintenance and wellbeing [3]. As a result, utilising a holistic approach to understand student health, which encompasses both physical and mental health, is imperative to fully understand its impact on education attainment outcomes. To this end, the current study conducts multilevel models to investigate the individual and collective role of students’ physical and mental health on post-primary attainment, specifically GCSE (General Certificate of Secondary Education) outcomes in Northern Ireland. In summary, GCSEs are standardised assessments that are completed in the final year of compulsory post-primary school across the UK. Students completing their GCSEs are typically 15–16 years old.

There are various pathways through which poor physical and/or mental health can impact upon educational outcomes including lower motivation to learn, lack of engagement with school and absenteeism [2, 4–7]. Students with physical or mental ill-health tend to report lower rates of connectivity with others in the school environment, higher exposure to bullying and fewer active coping mechanisms [5], all of which may play a contributory role in lower attainment.

Despite existing evidence highlighting the important role of health on educational outcomes, WHO emphasises the lack of disaggregated data in the international context on adolescent health according to intersecting inequalities such as socio-economic background and sex [1]. Gutman & Vorhaus [12] echoed this argument in the UK context, highlighting the little information known about the variation in the relationship between mental wellbeing and educational outcomes according to individual characteristics. However, the UK evidence base has since developed, with a greater understanding of the associations between mental health and educational outcomes (e.g., see [13]). Despite this, a lack of research remains in the UK jurisdiction of Northern Ireland, where this relationship has not been previously examined using linked administrative data. The exploration of disaggregated health data with education outcomes has been conducted in some countries but greater investigation is required, especially into the discourse that health and educational disparities are exacerbated by underlying factors such as poverty and sex [1, 2, 6]. The linking of disaggregated health and education data may highlight a complex reality, as factors such as family socio-economic position are strong predictors of educational outcomes among students with and without a chronic health condition [4]. However, depending upon the measure of health, these findings may vary. For example, young people from higher socio-economic backgrounds report better mental health, and boys tend to report better mental health than girls, with the latter being more likely to report multiple health issues [1]. This highlights the importance of examining how intersecting inequalities in education such as sex and socio-economic background interact independently, and together, with students’ physical and mental health to impact on education outcomes. Doing so provides an opportunity to understand whether specific subgroups of students are at particular risk of educational underachievement. The current study uses disaggregated, population wide administrative data from Northern Ireland to examine for the first time, the associations between students’ physical health, mental health and educational attainment outcomes, whilst accounting for their wider demographic profile such as socio-economic background, sex and ethnicity. The need for cross-departmental collaboration between health and education was recently outlined in the Northern Ireland Programme for Government 2024–2027 [14] and the Children and Young People’s Emotional Health and Wellbeing in Education Framework [15]. Northern Ireland, although apart of the UK, reflects a different socio-historical landscape than other regions in the UK. As a post-conflict society, studies have indicated intergenerational trauma of the conflict affecting the mental health of young people in the region [16]. This, coupled with the structure of the education system in Northern Ireland which differs from other UK regions, reiterates the importance of examining trends using local data to ensure future policy is informed with data that reflects the nuances of the region and experiences of young people residing there.

## Methods

### Data

The administrative dataset that provided the first record linkage between the household Census (2011), School Leavers Survey (2010–2014) and School Census (2010–2014) in Northern Ireland was used in this study to explore the associations between educational attainment, students’ physical health and students’ mental health. Informed consent to participate in the study was deemed unnecessary according to national regulations. The lawful basis for processing personal data falls under the Public Interest Article 6.1.E of the Data Protection Act 2018. These data were first used in a study conducted by Early et al. to examine the role of students’ socio-economic background, gender, religious affiliation and attended school on GCSE attainment outcomes [17]. The current analysis builds upon Early et al. [17] by focussing specifically on the associations between students’ health and their GCSE outcomes in the academic years 2010/2011, 2011/2012 and 2012/2013.

This study had three research questions:

1. Is educational attainment explained by the physical and mental health of a student?
2. Does physical health or mental health have a greater influence on educational attainment?
3. Is the educational attainment of particular groups of students affected by their physical health and mental health status?

Data from the three administrative sources were linked by the Northern Ireland Statistics and Research Agency (NISRA). The data were anonymised, held in a secure research environment and only made available for the purposes of this study. The linked data had a 93.3% match rate and 98.7% accuracy of matching Census records to students enrolled in the academic years 2010/11, 2011/12 and 2012/13. There was no significant bias between the sex of students or school year in the matched data. Although the most recent household Census was completed in 2021, these data are not yet linked to education data in Northern Ireland. As a result, this study used the most up to date linked education and Census data available for research. Using educational attainment data in the years immediately following the Census also ensured the most relevant socio-demographic information for each individual was used at these timepoints [17]. However, as these data are cross-sectional, the time lapsed between when the student completed their GCSEs and when the 2011 Census collected information about their socio-demographic profile is a limitation of the study. The dataset used for analysis cannot therefore account for whether there was a change in circumstances (including health status) between the 2011 Census and when the student completed their GCSEs.

This administrative dataset provides the first instance in which student attainment outcomes and health status have been linked in Northern Ireland. This provided a unique opportunity to explore the relative effect of physical health and mental health, whilst controlling for the wider socio-demographic profile of students and their parents, as previously examined by Early et al. [17]. This is the first study in which the impact of students’ physical and mental health on their educational attainment outcomes are considered in the one study using complex statistical analysis and population wide data in Northern Ireland.

The household Census from 2011 included questions about the health status of individuals which are utilised in this study. Although these questions are centred around the health of students, it is usually the parent/carer in the household where a young person resides that completes the Census. As a result, these measures, although referring to a student’s health, are likely to be parent/carer-reported rather than self-reported by the young person. Questions in the Census that address the health of respondents varies between UK nations. Although there is consistency in the measures of self-report health status and presence of a limiting illness, only respondents in Scotland and Northern Ireland are asked about the presence of a specific physical health condition or mental health condition. More specifically, the household Census in Northern Ireland provides greater disaggregation of the physical health conditions an individual can select from, and the category of mental health condition also includes emotional or psychological conditions. By way of comparison, the Scottish Census has nine categories referring to physical and mental health conditions, whilst the Census in Northern Ireland has 12 for the same question.

### Method

This current study closely follows the methodology executed by Early et al. [17]. Cross-sectional data is utilised for three complete student cohorts, aged 15–16 years, who completed their GCSEs in 2010/2011 (*n* = 21,048), 2011/2012 (*n* = 20,551) and 2012/2013 (*n* = 19,774). Overall, 61,373 students and 217 schools were included in the analysis. Multilevel models, specifically random intercept models, were conducted using Stata 16 to account for the nested data structure. In addition, five interaction terms were tested to determine the influence of health status and demographic characteristics such as sex and socio-economic background (measured according to Free School Meal Eligibility [FSME]) on attainment outcomes. Interaction terms were created by multiplying two binary variables together (outlined below).

The following interaction terms were tested and those with an asterisk (*) were statistically significant:

- Sex and physical health condition*
- Sex and mental health condition*
- FSME and physical health condition*
- FSME and mental health condition
- Physical health condition and mental health condition*

### Outcome measure

This study used the same continuous outcome measure as Early et al. [17] to ensure consistency and comparability, whilst also ensuring the measure of academic attainment was inclusive, measured on its natural continuum and reflected changes to numeric grading (from alphabetical grading) across the UK. For this study, an overall GCSE score was computed using data from the School Leavers Survey and was inclusive of the alphabetical grades A* - U. Each alphabetical grade was assigned a numeric value ranging from 9 to 1. The highest alphabetical grade was assigned the highest numeric value (A* = 9), and the lowest alphabetical grade was assigned the lowest numeric value (U = 1). Each alphabetical grade was multiplied with its assigned numeric value to compute an overall GCSE score for each student based on how many GCSEs they achieved at each alphabetical grade. Table 1 provides the mean GCSE score for each cohort.

**Table 1:** Frequencies and mean GCSE score for idividual- and school-level independent variables

Variable	*N* (%)	Mean GCSE Score (Standard Deviation)
Cohort		
Cohort 1 (2010/11)	21,048 (34.3%)	51.4 (23.4)
Cohort 2 (2011/12)	20,551 (33.5%)	52.2 (22.9)
Cohort 3 (2012/13)	19,774 (32.2%)	51.6 (23.7)
Household Structure		
Mother resides in same household	57,778 (94.1%)	52.4 (23.1)
Mother does not reside in same household	3,595 (5.9%)	41.2 (24.3)
Father resides in same household	42,297 (68.9%)	56.1 (22.0)
Father does not reside in same household	19,076 (31.1%)	42.1 (23.4)
Sex		
Male	31,352 (51.1%)	48.4 (23.7)
Female	30,021 (48.9%)	55.2 (22.5)
Birth Month		
January– March	15,008 (24.5%)	51.0 (23.4)
April - June	15,472 (25.2%)	50.6 (23.3)
July– September	15,783 (25.7%)	53.2 (23.3)
October– December	15,018 (24.5%)	52.1 (23.4)
Month unknown	92 (0.1%)	44.2 (22.4)
Religion		
Catholic	27,584 (44.9%)	54.0 (23.2)
Protestant	21,035 (34.3%)	49.9 (23.0)
Other religion	3,884 (6.3%)	55.1 (22.0)
No religion	5,367 (8.7%)	51.7 (22.8)
Religion not stated	3,503 (5.7%)	41.2 (25.1)
Ethnicity		
White	60,043 (98.4%)	51.6 (23.3)
Chinese	507 (0.8%)	59.4 (23.2)
Black	100 (0.2%)	44.0 (21.6)
Mixed	314 (0.5%)	58.3 (23.2)
Other	49 (0.1%)	58.1 (24.5)
First Language		
English is first language	59,828 (97.5%)	51.9 (23.3)
English is not first language	1,545 (2.5%)	46.6 (23.5)
Country of Birth		
Northern Ireland	57,242 (93.3%)	51.7 (23.3)
United Kingdom	2,004 (3.3%)	54.3 (22.8)
Republic of Ireland	311 (0.5%)	53.6 (24.3)
Europe	931 (1.5%)	43.2 (23.6)
Other	885 (1.4%)	58.7 (22.3)
Carer Status		
No caring responsibilities	58,548 (95.4%)	51.7 (23.4)
1–19 h per week	2,286 (3.7%)	53.8 (22.2)
20 + hours per week	539 (0.9%)	46.7 (23.7)
Self-report health status		
Very good health	46,138 (75.2%)	53.9 (22.7)
Good health	13,155 (21.4%)	46.7 (23.6)
Fair health	1,799 (2.9%)	36.0 (24.8)
Bad health	222 (0.4%)	34.1 (25.6)
Very bad health	59 (0.1%)	30.1 (22.4)
Presence of an illness or disability that limits daily activities		
Not limited	58,084 (94.6%)	52.5 (23.0)
Limited a little	2,242 (3.6%)	41.3 (25.2)
Limited a lot	1,047 (1.7%)	30.0 (23.9)
Physical Condition		
No physical health condition	52,519 (85.6%)	53.0 (22.8)
Has a physical health condition	8,854 (14.4%)	44.1 (25.1)
Mental Health Condition		
No mental health condition	60,992 (99.3%)	51.9 (23.3)
Has a mental health condition	451 (0.7%)	30.7 (25.0)
Free School Meal Eligibility		
Not eligible for FSM	50,476 (82.2%)	55.2 (22.0)
Eligible for FSM	10,897 (17.8%)	35.6 (22.7)
Mother’s qualifications		
Degree level qualification	15,685 (27.1%)	64.8 (19.7)
School level qualification	29,347 (50.8%)	51.7 (21.5)
Other qualification	2,032 (3.5%)	45.8 (22.3)
No qualification	10,714 (18.5%)	37.4 (22.4)
Father’s qualifications		
Degree level qualification	11,169 (26.4%)	67.7 (18.6)
School level qualification	15,683 (37.1%)	55.6 (20.5)
Other qualification	6,299 (14.9%)	52.7 (20.9)
No qualification	9,146 (21.6%)	45.2 (22.3)
Mother’s Occupational Status		
Professional	14,097 (25.8%)	63.1 (20.3)
Intermediate	19,597 (33.9)	55.4 (21.1)
Semi-routine/routine	17,920 (31.0%)	44.6 (22.2)
Unemployed	5,354 (9.3%)	37.7 (23.8)
Father’s Occupational Status		
Professional	12,525 (29.6)	65.9 (19.1)
Intermediate	18,461 (43.6%)	55.3 (20.9)
Semi-routine/routine	9,422 (22.3%)	47.2 (21.8)
Unemployed	1,889 (4.5%)	43.4 (24.0)
Housing Tenure		
Privately owned	46,767 (76.2%)	56.3 (21.5)
Privately rented	6,308 (10.3%)	40.1 (22.9)
Rented from Housing Association or Executive	7,374 (12.0%)	33.4 (21.8)
Other tenure	924 (1.5%)	44.7 (25.5)
Property Value		
> £200,000	13,473 (21.9%)	63.8 (19.4)
£150,001 - £200,000	13,547 (22.1%)	56.7 (20.7)
£100,001 - £150,000	20,999 (34.2%)	46.6 (22.5)
≤ £100,000	10,980 (17.9%)	40.3 (24.2)
Value unknown	2,374 (3.9%)	53.2 (22.9)
Northern Ireland Multiple Deprivation Measure (2010) - Income		
10– Least Deprived Residential Decile	5,675 (9.2%)	63.2 (20.6)
9	6,253 (10.2%)	58.1 (21.0)
8	6,425 (10.5%)	56.3 (21.8)
7	6,875 (11.2%)	54.7 (22.0)
6	6,786 (11.1%)	53.5 (22.3)
5	6,230 (10.1%)	51.5 (22.6)
4	6,130 (10.0%)	49.6 (23.2)
3	5,702 (9.3%)	46.4 (23.8)
2	5,767 (9.4%)	43.9 (23.7)
1– Most Deprived Residential Decile	5,530 (9.0%)	39.2 (23.5)
School Type		
Non-grammar	36,809 (60.0%)	39.8 (20.5)
Grammar	24,564 (40.0%)	69.7 (14.2)
School Management Structure		
Controlled	20,877 (34.0%)	45.8 (23.0)
Catholic Maintained	16,586 (27.0%)	42.3 (21.1)
Voluntary	18,393 (30.0%)	70.4 (14.0)
Other Maintained	179 (0.3%)	46.1 (15.5)
Integrated	5,338 (8.7%)	39.9 (19.2)

### Predictor variables

Previous research has highlighted that a range of socio-demographic factors such as sex, socio-economic background, parental education and parental occupation have known relationships with educational attainment [17]. As a result, it is important these factors are accounted for when assessing the impact of an individual’s physical and mental health on their attainment [11].

To aid comparability between the current study and Early et al. [17], the multilevel models conducted for this study included the same individual-level predictor variables of sex, religious affiliation and socio-economic background (FSME, mothers’ qualifications, fathers’ qualifications, mothers’ occupational status, fathers’ occupational status, housing tenure, property value and the Northern Ireland Multiple Deprivation Measure [NI-MDM, 2010] for income). However, a range of additional student-level variables were also accounted for in this current analysis that were not included in the study by Early et al. [17]. The additional variables controlled for in the current study are students’ month of birth, ethnicity, migrant status, carer status and health status. Migrant status was examined through two proxies: country of birth (Northern Ireland, UK, EU, Republic of Ireland or other) and whether English was a first language (yes or no). Carer status considered whether a student was a carer and whether they provided 1 to 19 h of care per week, 20 or more hours per week, or none.

A holistic approach to understanding the influence of health on educational outcomes was adopted in this study through the inclusion of four measures. Physical health was examined through three variables.

- Firstly, self-reported health status derived from the Census question *how is your health in general.* This variable had five answer categories ranging from very good to very bad. Very good health was the reference category in the multilevel model.
- Secondly, the measure of whether a limiting health problem or disability was present refers to the Census question *are your day-to-day activities limited because of a health problem or disability which has lasted*,* or is expected to last*,* at least 12 months*. Presence of a limiting health problem or disability consisted of three answer categories: limited a lot by a health problem/disability, limited a little by a health problem/disability and no health problem/disability. No health problem/disability was the reference category in the multilevel model analysis.
- Finally, the Census question *do you have any of the following conditions which have lasted*,* or are expected to last*,* at least 12 months* was recoded into a binary variable to indicate the presence or absence of a *physical* condition.

Mental health was measured using one variable. The Census question *do you have any of the following conditions which have lasted*,* or are expected to last*,* at least 12 months* allowed participants to indicate whether they experienced an *‘emotional*,* psychological or mental health condition’*. The yes/no answer to this question (separated out from the other physical conditions listed) was used to investigate the relative impact of students’ mental health on education outcomes. This study considers the presence, severity and chronicity of physical health conditions and mental health conditions separately rather than through a composite measure. This was based on the rationale that each variable contributed different and important information to understand the impact of students’ health on their educational attainment outcomes. However, this may also be viewed, and is recognised, as a potential limitation of the analytical approach of this study.

**Fig. 1:**
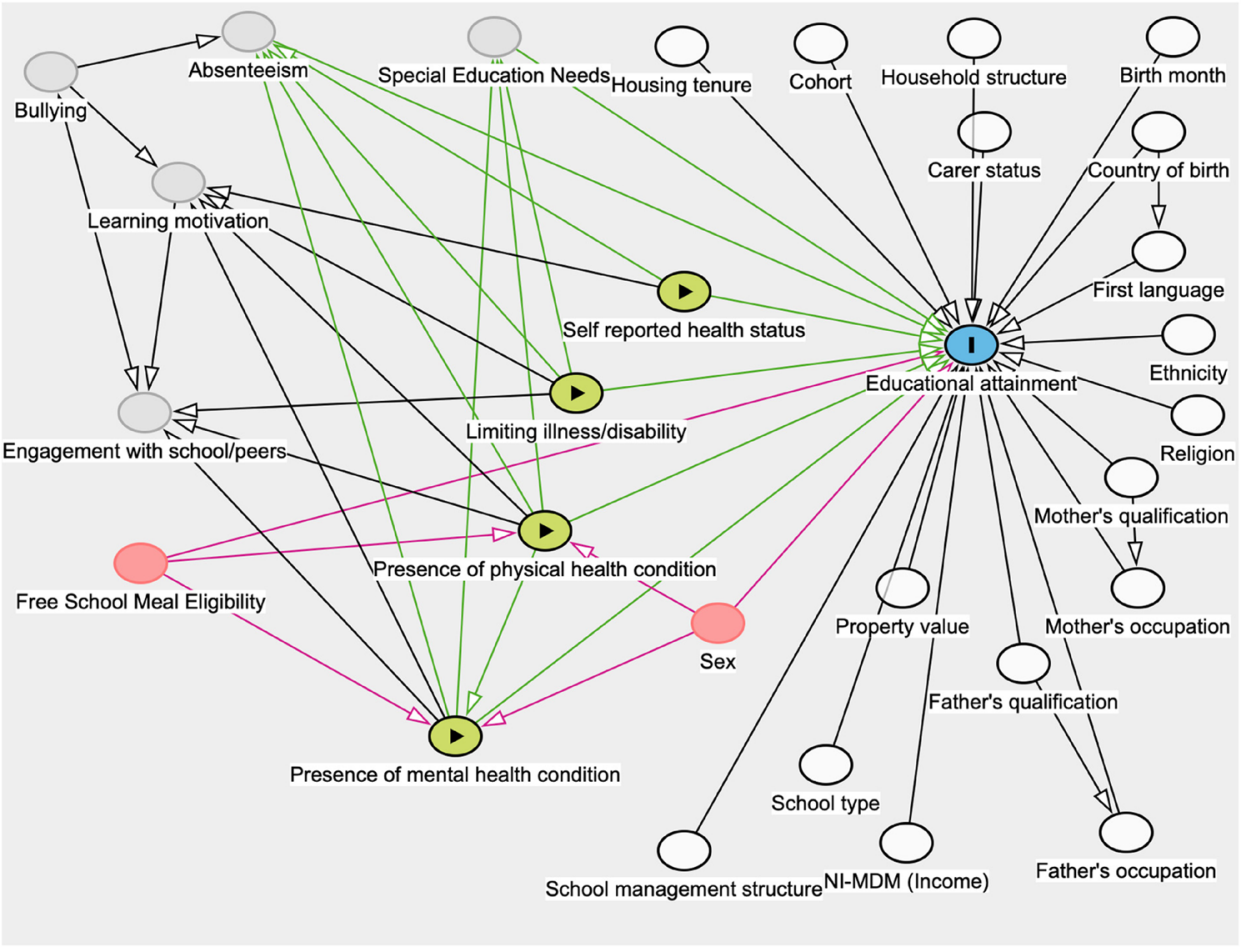
Directed Acyclic Graph (DAG) of the Conceptual Model legend: Outcome variable;  Exposure/independent variables;  Confounding variables; Adjusted variables;  Unobserved variables; Causal path;  Pathway involving a confounding variable

At the school-level, school type and school management structure accounted for the academically and religiously segregated education system in Northern Ireland. School type was a binary variable referring to grammar schools and non-grammar schools. School management structure had five categories (voluntary, integrated, controlled, catholic maintained and other maintained schools). Figure 1 presents a Directed Acyclic Graph (DAG) to illustrate the conceptual model^1^. In Fig. 1, the four variables measuring students’ physical and mental health are categorised as exposure (independent) variables. All other independent variables included in the multilevel model are categorised as co-variates that are adjusted for. This is not because these variables are less important or influential on the outcome but because these have been previously discussed [17] and are not central to the research questions of the current study. Two variables (sex and FSME) are categorised as confounding variables in the DAG as although these factors may affect the outcome measure directly, the current analysis is predominantly interested in their interactions with the presence of physical and/or mental health conditions.

## Results

The analysis of this study presents the multilevel models and interaction terms that examine the associations between the student-level health measures, students’ demographic profile and GCSE attainment outcomes. The main multilevel model is presented in Table 2 and addresses research questions 1 and 2^2^. Descriptive statistics for each predictor variable are provided in Table 1.

**Table 2:** Multilevel model examining GCSE score according to individual- and school-level characteristics in Northern Ireland

Number of Students: 61,373 Number of Schools: 217 Log likelihood: −254612.59		
**Variable**	**β (SE)**	**Cohen’s** ***d*** **(95% Confidence Intervals)**
Cohort *(reference: Cohort 1)*		
Cohort 2	0.44 (0.15)	0.03 (0.01, 0.05)
Cohort 3	0.19 (0.15)	0.01 (−0.01, 0.03)
No maternal data *(reference: maternal data provided)*	−7.37 (0.31)	−0.48 (−0.52, −0.45)
No paternal data *(reference: paternal data provided)*	−6.12 (0.23)	−0.62 (−0.64, −0.61)
Birth Month *(reference: July– September)*		
October - December	−0.29 (0.17)	−0.05 (−0.07, −0.05)
January - March	−1.00 (0.17)	−0.09 (−0.12, −0.07)
April - June	−1.23 (0.17)	−0.11 (−0.13, −0.09)
Unknown month	−1.96 (1.60)	−0.39 (−0.60, −0.18)
Sex *(reference: male)*		
Female	6.22 (0.14)	0.29 (0.28, 0.31)
Religion *(reference: Catholic)*		
Protestant	0.38 (0.27)	−0.18 (−0.19, −0.16)
Other religion	1.30 (0.35)	0.05 (0.01, 0.08)
No religion	0.47 (0.31)	−0.10 (−0.13, −0.07)
Not stated	−4.15 (0.30)	−0.55 (−0.58, −0.51)
Ethnicity *(reference: White)*		
Chinese	3.20 (0.88)	0.33 (0.25, 0.42)
Black	−0.42 (1.60)	−0.33 (−0.52, −0.13)
Mixed	2.52 (0.87)	0.29 (0.18, 0.40)
Other	−0.61 (2.20)	0.28 (−0.001, 0.56)
English as First Language *(reference: English is not first language)*	−0.22 (0.71)	−0.23 (0.18, 0.28)
Country of Birth *(reference: Northern Ireland)*		
UK	0.48 (0.35)	0.11 (0.07, 0.16)
Republic of Ireland	1.13 (0.87)	0.08 (−0.03, 0.19)
EU	1.87 (0.78)	−0.36 (−0.43, −0.30)
Other	3.14 (0.61)	0.30 (0.23, 0.37)
Carer Status *(reference: no caring responsibilities)*		
Provides 1–19 h of care per week	1.16 (0.33)	0.09 (0.05, 0.13)
Provides 20 + hours of care per week	1.45 (0.66)	−0.21 (−0.30, −0.13)
Self-report Health Status *(reference: very good health)*		
Good health	−1.31 (0.16)	−0.28 (−0.30, −0.26)
Fair health	−3.35 (0.42)	0.75 (−0.79, −0.70)
Bad health	−2.91 (1.11)	−0.84 (−0.97, −0.70)
Very bad health	−3.22 (2.03)	−1.01 (−1.26, −0.75)
Presence of an illness/disability that limits daily activities *(reference: not limited)*		
Limited a little	−1.91 (0.39)	−0.48 (−0.53, −0.44)
Limited a lot	−7.42 (0.57)	−0.98 (−1.04, −0.92)
Physical Condition *(reference: no physical health condition)*		
Has a physical health condition	−1.69 (0.22)	−0.39 (−0.41, −0.36)
Mental Health Condition *(reference: no mental health condition)*		
Has a mental health condition	−6.22 (0.75)	−0.91 (−1.00, −0.82)
Free School Meal Eligibility *(reference: not eligible for FSM)*		
Eligible for FSM	−3.19 (0.21)	−0.89 (−0.91, −0.86)
Mother’s qualifications *(reference: degree level)*		
School level qualification	−2.00 (0.19)	−0.63 (−0.65, −0.61)
Other qualification	−3.76 (0.40)	−0.95 (−1.00, −0.90)
No qualification	−5.52 (0.26)	−1.31 (−1.34, −1.29)
Father’s qualifications *(reference: degree level)*		
School level qualification	−1.70 (0.22)	−0.61 (−0.64, −0.59)
Other qualification	−2.57 (0.28)	−0.77 (−0.80, −0.74)
No qualification	−3.56 (0.27)	−1.11 (−1.13, −1.08)
Mother’s Occupational Status *(reference: professional)*		
Intermediate	0.02 (0.19)	−0.38 (−0.39, −0.35)
Semi-routine/routine	−1.25 (0.22)	−0.86 (−0.89, −0.84)
Unemployed	−2.88 (0.29)	−1.19 (−1.22, −1.16)
Father’s Occupational Status *(reference: professional)*		
Intermediate	−1.04 (0.21)	−0.52 (−0.55, −0.50)
Semi-routine/routine	−1.78 (0.25)	−0.92 (−0.95, −0.89)
Unemployed	−1.60 (0.41)	−1.14 (−1.19, −1.08)
Housing Tenure *(reference: rented from Housing Association or Executive)*		
Privately owned	3.80 (0.24)	1.06 (1.04, 1.09)
Privately rented	0.32 (0.28)	0.30 (0.27, 0.33)
Other tenure	0.93 (0.54)	0.51 (0.44, 0.58)
Property Value *(reference: > £200*,*000)*		
£150,001 - £200,000	−0.46 (0.19)	−0.35 (−0.38, −0.33)
£100,001 - £150,000	−1.80 (0.19)	−0.81 (−0.83, −0.78)
≤ £100,000	−2.52 (0.24)	−1.08 (−1.11, −1.06)
Value unknown	−1.39 (0.35)	−0.53 (−0.57, −0.49)
Northern Ireland Multiple Deprivation Measure (2010) - Income	0.16 (0.03)	**-**
School Type *(reference: non-grammar)*		
Grammar	23.79 (1.73)	1.64 (1.62, 1.66)
School Management Structure *(reference: Voluntary)*		
Controlled	−3.75 (1.75)	−1.27 (−1.29, −1.25)
Catholic Maintained	1.95 (2.07)	−1.58 (−1.61, −1.56)
Other Maintained	8.08 (6.61)	−1.73 (−1.88, −1.59)
Integrated	−1.28 (2.39)	−1.99 (−2.03, −1.95)

*Outcome measure*: GCSE score

Whilst the analysis of the current study is based on the same dataset as Early et al. [17], the magnitudes of the effects differ because the models include different independent variables. The same direction of effects is evident for most variables across both studies. For example, the current analysis indicates that female students achieve higher GCSE scores than their male peers (b = 6.22, *p* < 0.001; *d =* 0.29, 95% CI: 0.28, 0.31). In addition, the lower GCSE attainment of students eligible for Free School Meals (FSM) compared to their non-eligible peers is evident (b = −3.19, *p* < 0.001; *d =* −0.89, 95% CI: −0.91, −0.86). However, differences in the direction of effects in other variables are due to the inclusion of a greater number of covariates in the current analysis; some of which were not previously accounted for but are controlled for in this study.

### Self-report health status

Students reporting very good health achieved the highest GCSE scores. Compared to these students, lower GCSE scores were achieved for students reporting:

- good health (b = −1.31, *p* < 0.001; *d =* −0.28, 95% CI: −0.30, −0.26).
- fair health (b = −3.35, *p* < 0.001; *d =* −0.75, 95% CI: −0.79, −0.70).
- bad health (b = −2.91, *p* < 0.01; *d =* −0.84, 95% CI: −0.97, −0.70) or
- very bad health (b = −3.22, *p* < 0.01; *d =* −1.01, 95% CI: −1.26, −0.75).

Based on Cohen’s *d*, the smallest attainment difference across the health measures in the analysis is between those reporting very good health and good health, whilst the largest difference is between students reporting very good health and very bad health. A positive association between self-reported health and GCSE attainment is therefore evident, i.e. very good health is associated with higher GCSE scores and very bad health is associated with lower GCSE scores.

### Presence of a limiting health problem or disability

Students who reported having a health problem or disability that limited their daily activities a lot had substantially lower GCSE scores than their peers who reported no limiting health problem or disability (b = −7.42, *p* < 0.001; *d =* −0.98, 95% CI: −1.04, −0.92). This was the second largest effect (based on Cohen’s *d*) among the health measures included in the multilevel model. The impact of having a health problem or disability that limited daily activity a little was lower but is still of significance (b = −1.91, *p* < 0.001; *d =* −0.48, 95% CI: −0.53, −0.44). In summary, having a health problem or disability that limits daily activity is associated with lower GCSE scores.

### Presence of a physical health condition

The presence of a physical health condition reflected a significant association with GCSE attainment, even when controlling for students’ self-reported health status, a limiting health problem/disability and a mental health condition. Students with a physical health condition had lower GCSE scores than students with no physical health condition (b = −1.69, *p* < 0.001; *d =* −0.39, 95% CI: −0.41, −0.36). A positive association between better physical health and higher GCSE attainment is therefore evident, with those reporting poorer physical health (through the presence of a health condition) having poorer GCSE outcomes.

### Presence of a mental health condition

The presence of a mental health condition had one of the largest impacts among the health variables on GCSE attainment outcomes in the model. Students reporting the presence of an emotional, psychological or mental health condition had substantially lower GCSE scores than their peers with no mental health condition (b = −6.22, *p* < 0.001; *d =* −0.91, 95% CI: −1.00, −0.82). A positive association between good mental health and higher GCSE outcomes is therefore apparent, i.e., good mental health is associated with higher GCSE scores and poor mental health (measured through the presence of a mental health condition) is associated with lower GCSE scores.

### Interactions

To address research question 3, interaction terms were added (one at a time) to the main model above. In total, five interactions were tested, four of which reflected statistical significance: sex and physical health condition; sex and mental health condition; FSME and physical health condition, and physical health condition and mental health condition.

**Fig. 2:**
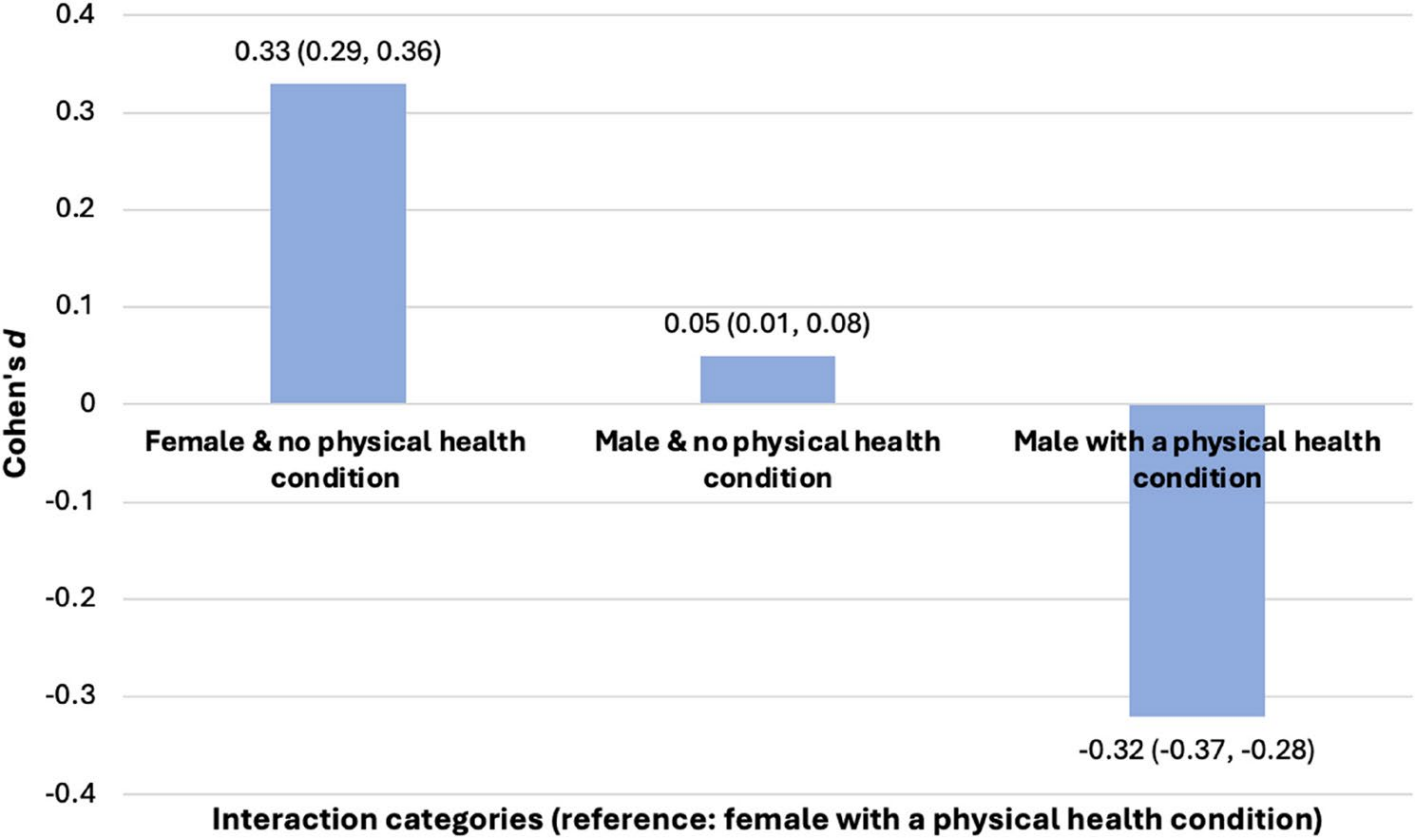
Differences in mean GCSE scores according to students’ sex and presence of a physical health condition

When interacting students’ sex and the presence of a physical health condition, females who had no physical health condition achieved higher GCSE attainment scores than females with a physical health condition (*d =* 0.33, 95% CI: 0.29, 0.36). This direction of effect was also evident with male students who had no physical health condition but to a lesser extent (*d* = 0.05, 95% CI: 0.01, 0.08). In contrast, male students with a physical health condition had lower GCSE attainment scores than females with a physical health condition (*d* = −0.32, 95% CI: −0.37, −0.28) (Fig. 2).

**Fig. 3:**
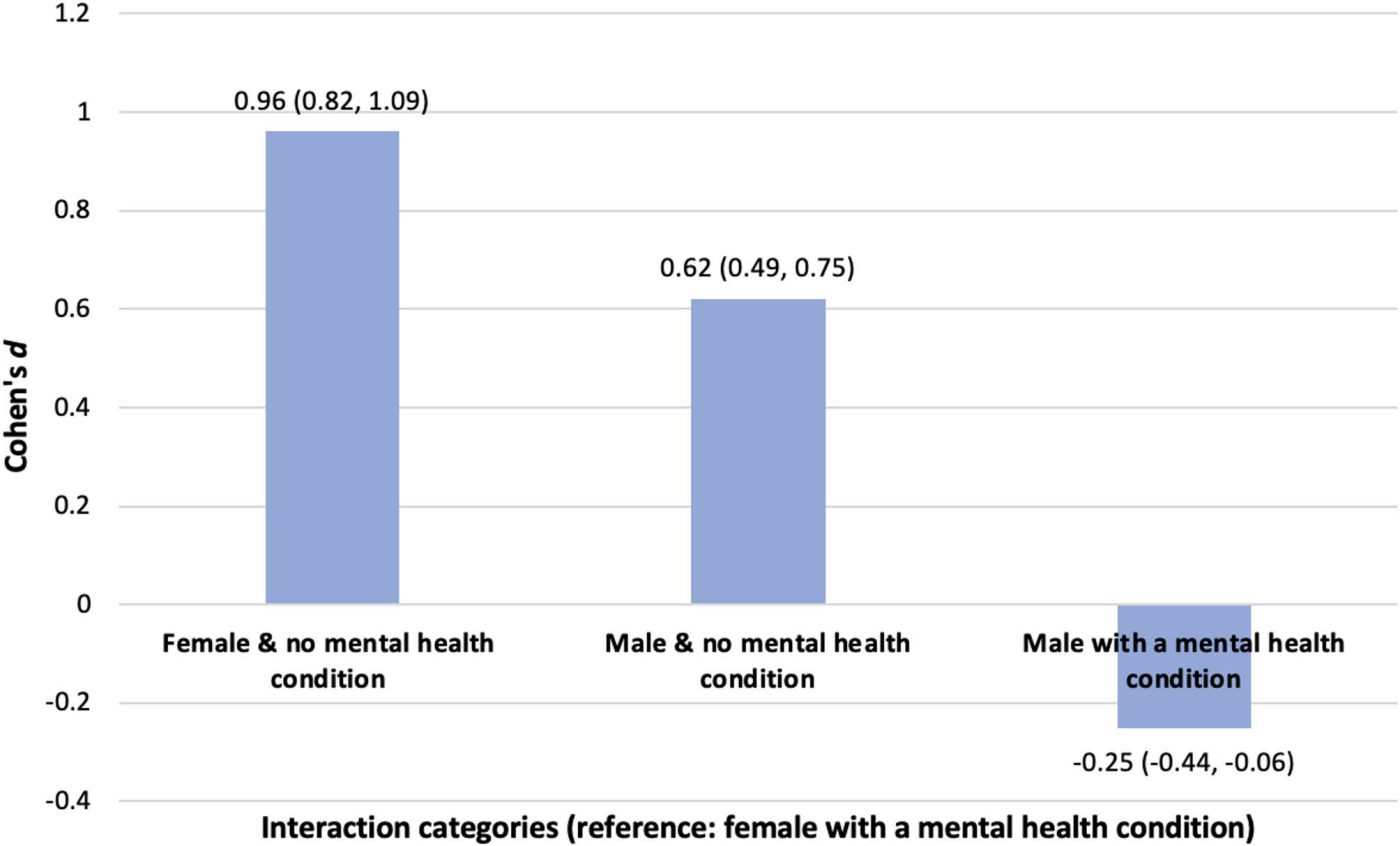
Differences in mean GCSE scores according to students’ sex and presence of a mental health condition

The interaction between sex and the presence of a mental health condition indicated the higher GCSE attainment of both sexes with no mental health condition (Fig. 3). The largest difference was evident between females with no mental health condition and females with a mental health condition, with the former group having higher GCSE scores (*d* = 0.96, 95% CI: 0.82, 1.09). Males with no mental health condition also had higher attainment than females with a mental health condition (*d* = 0.62, 95% CI: 0.49, 0.75). However, males with a mental health condition had lower GCSE attainment than their female peers with a condition (*d* = −0.25, 95% CI: −0.44, −0.06).

**Fig. 4:**
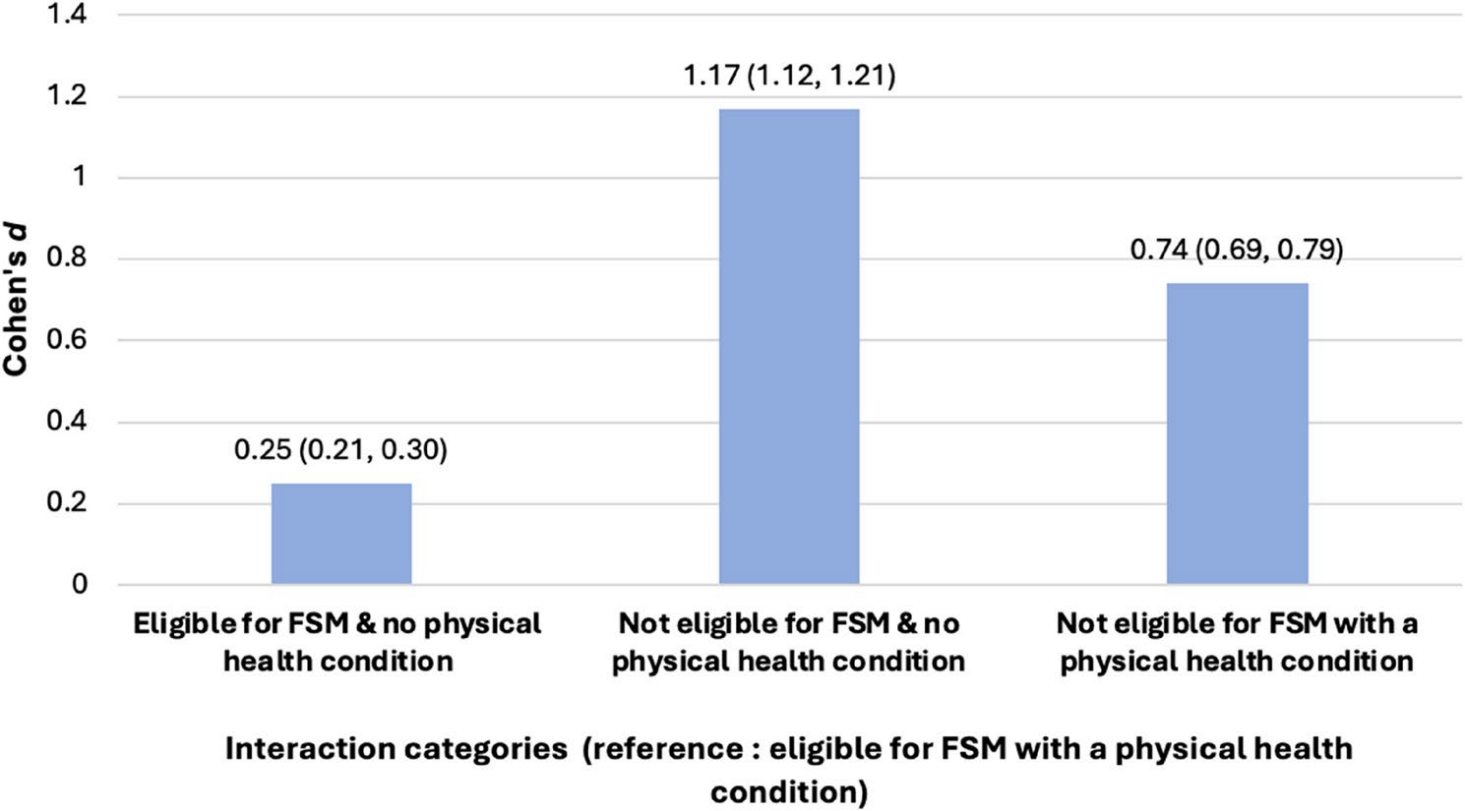
Differences in mean GCSE scores according to FSME and presence of a physical health condition

The interaction of a physical health condition and a student’s socio-economic background (based on the proxy indicator of FSME) also reflected statistical significance (Fig. 4). Those students eligible for FSM with a physical health condition achieved lower GCSE scores when compared to students who were:

- eligible for FSM with no physical health condition (*d* = 0.25, 95% CI: 0.21, 0.30).
- not eligible for FSM with a physical health condition (*d* = 0.74, 95% CI: 0.69, 0.79).
- not eligible for FSM with no physical health condition (*d =* 1.17, 95% CI: 1.12, 1.21).

The presence of a physical health condition *and* mental health condition were considered to utilise a holistic approach in understanding the impact of student health on education outcomes. The interaction reflected statistical significance and indicated the lower GCSE attainment of students with a physical health condition and mental health condition compared to those with:

- a physical health condition but no mental health condition (*d *= 0.64, 95% CI: 0.52, 0.76).
- no physical health condition but a mental health condition (*d* = 0.22, 95% CI: 0.02, 0.41).
- neither a physical or mental health condition (*d *= 1.07, 95% CI: 0.96, 1.19).

**Fig. 5:**
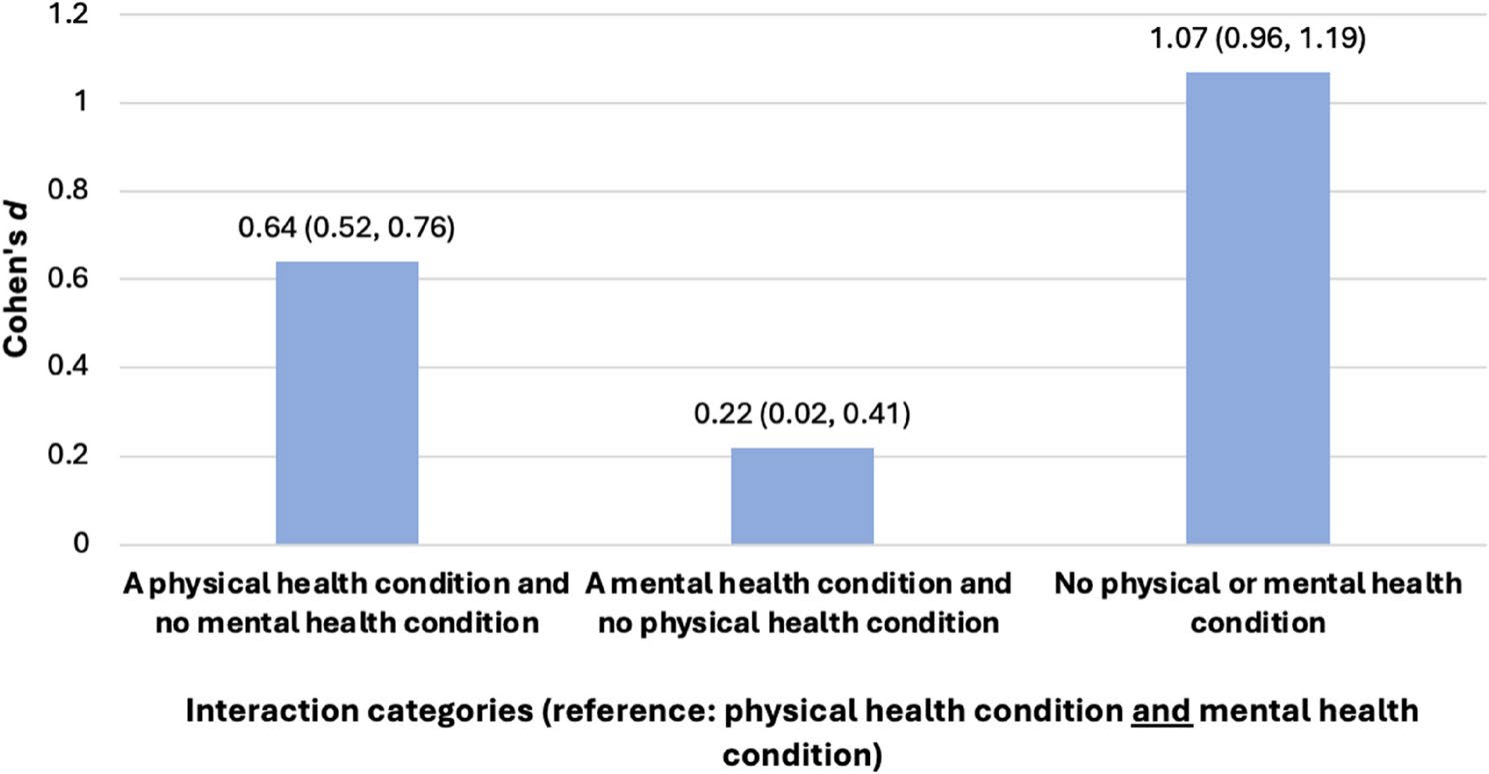
Differences in mean GCSE scores according to the presence of a physical health condition *and* mental health condition

This inclusive approach to measuring student health highlights that the largest attainment gap is between those with both a physical and mental health condition and those with neither (Fig. 5).

## Discussion

This study found that educational attainment is influenced by both the physical and mental health of a student, thus addressing research question 1. Across all health indicators included in the analysis, a positive association between poorer health and lower GCSE outcomes was evident for students aged 15–16 years old in Northern Ireland.

### Physical health

As students report poor physical health, their GCSE attainment also becomes poorer. The largest difference was evident in the measure of self-report health status, specifically between students who reported *very bad health* and *very good health (d* = −1.01, 95% CI: −1.26, −0.75). This variable clearly illustrates how the grading of self-reported health status impacts educational outcomes. For example, those with *good health* had lower GCSE outcomes than students with *very good health* (*d* = −0.28, 95% CI: −0.30, −0.26). A positive association between *very good health* and higher GCSE scores is therefore evident, highlighting the important role of health status grading for our understanding and meaningful interpretation of how health impacts educational outcomes.

The overall measurement of self-reported health must be acknowledged as it could be deemed a relatively blunt indicator to understand a complex phenomenon. For example, the Census questions ask respondents *how is your health in general?* This measure is dependent upon an individual’s interpretation of health and could include perceptions of their physical health, mental health, or both. In addition, it may be the parent/carer of a young person who is completing the household Census on their child’s behalf. An individual’s interpretation of how to categorise their own health is subjective [18, 19], with variations reported in the approach of different social groups [20]. For example, previous research has suggested that only those with severe conditions, illnesses or disabilities are likely to define their health as poor [21]. This may be confirmed in the current study as only a small proportion of students (0.1%) report *very bad health*. Despite the limitations of this measure, a stark effect was observed in the analysis as students with *very bad health* achieved the lowest GCSE outcomes compared to those with *very good health*.

An association between a limiting health problem or disability and GCSE attainment was also evident. Students who were limited a lot by a health problem or disability achieved lower GCSE scores than those with no limitations (*d* = −0.98, 95% CI: −1.04, −0.92). However, what constitutes as *limited a little* and *limited a lot* by a health problem or disability should be considered. As above, this interpretation is subjective to an individual. As the Census question asks about health problems or disabilities that have lasted, or are expected to last, at least 12 months, it is likely those who are limited a lot represent students with the most severe health problems or disabilities. For example, only 1.7% of students reported being *limited a lot* in the 2011 household Census. It should also be acknowledged that some individuals may mask their limitations based on a health condition or disability to reduce stigma within social institutions such as education.

The findings from this current study on the influence of physical health on educational outcomes reiterate previous research in the UK and international context which highlights students with better health are likely to have higher academic attainment [22, 23]. One explanation suggests students with better physical health have the ability to benefit from high quality teaching through regular school attendance, which assists their ability in reaching their academic potential [12]. On the other hand, studies have suggested school attendance, concentration and an individual’s mental health can be negatively affected by poor physical health, which in turn can impact upon educational outcomes [3].

### Mental health

The current study, along with previous evidence, affirms that poor mental health is associated with lower educational outcomes [12, 24], even after accounting for other explanatory indicators such as socio-economic background, parenting and school-level factors [11, 13]. However, the measure of mental health in this study is a blunt indicator which only asks respondents to report upon conditions *that have lasted*,* or are expected to last*,* at least 12 months.* This wording therefore suggests that only relatively severe cases of mental ill-health are captured, as evidenced by only 0.7% of students reporting a mental health condition in the 2011 Census. This is likely to be an underestimation of mental health issues among the 16-year-old population in Northern Ireland. For example, other prevalence data such as the Youth Wellbeing Prevalence Survey suggests higher rates of mental health conditions among children and young people in Northern Ireland [25]. However, the Youth Wellbeing Prevalence Survey provides a more granular exploration of specific mental health conditions than what is available in the household Census. Furthermore, the likely underestimation of mental health issues among 16-year-olds in the current study may be impacted by the fear of stigma and negative attitudes if they report having a mental health condition [26, 27].

Determining whether a student’s physical health or mental health has a greater influence on their educational attainment (research question 2) is dependent upon the indicator used. If the focus is upon the presence of a physical health condition or mental health condition (that has lasted, or is expected to last, at least 12 months), it is important to highlight the low proportion of students reporting a physical health condition (14.4%) or mental health condition (0.7%). Although these are blunt measures of physical and mental health, the analysis indicates that while both are influential on attainment outcomes, the presence of an *emotional*,* psychological or mental health condition* had a greater impact, with a stark difference observed. In summary, although the presence of a physical health condition negatively affected GCSE outcomes (*d* = −0.39, 95% CI: −0.41, −0.36), the impact of a mental health condition was much higher (*d* = −0.91, 95% CI: −1.00, −0.82).

### Physical health and mental health

The influence of physical and mental health should be considered separately but also collectively to understand their reciprocal relationship [28]. The effect of physical and mental health conditions on attainment was accounted for in this study through an interaction of having a physical *and* mental health condition. The analysis, in some way, indicates a double disadvantage for students with a physical *and* mental health condition. For example, students with a physical *and* mental health condition had lower GCSE attainment than those with only a physical health condition or mental health condition, or none.

The attainment of particular social groups (based on socio-economic background and sex) also varied according to their physical health and mental health (research question 3). Overall, students who are not eligible for FSM have higher attainment, regardless of physical or mental health status. Within the group with *no* physical health conditions, females achieved higher grades than males. Of those students *with* a physical condition, females continued to achieve higher GCSE grades than their male counterparts. Interestingly the attainment gap between *females with* a health condition and *males without* a health condition, was in favour of the latter (as one might expect), but it was only a very small difference (*d* = 0.05, 95% CI: 0.01, 0.08).

Similar trends were evident in the interaction between sex and the presence of a mental health condition. Students with *no mental health* condition achieved better GCSE results compared to students *with a mental health condition* overall. Within the *no mental health* condition females outperformed males, and within the group of students who *did* report a mental health condition, females also outperformed males, after controlling for other explanatory factors (*d* = −0.25, 95% CI: −0.44, −0.06). Other studies have found similar trends [8, 11, 13]. For example, in England, Smith et al. [11] reported males and females with mental health difficulties were less likely to achieve the benchmark of five or more GCSEs A*-C, including English and mathematics. However, when controlling for other explanatory factors in their analysis, this relationship only remained significant for males [11]. Some studies have highlighted that trends in educational attainment vary according to the mental health condition/illness [8]. Although the present study does not look at specific mental health conditions due to the aggregation of data in the 2011 Census of the population and the available indicators being somewhat blunt measures, it is nonetheless the first analysis of its kind that reports on data from Northern Ireland. Finally, despite the known association between socio-economic background and health, the interaction between FSME and a mental health condition was not statistically significant in this study. Though some studies have highlighted that the association between poorer mental health and lower educational attainment transcends across socio-economic categories [11].

### Implications for policy

Students with a physical and/or mental health condition may experience cumulative disadvantage over time. This may be reflected through consistent absenteeism and academic underachievement across the education system. However, due to the lack of available education data for research purposes at earlier timepoints of the compulsory education system in Northern Ireland (for example, primary school), it is difficult to understand, if and when, this cumulative disadvantage emerges, along with when the most effective time for interventions would be. Consequently, greater data access is required, along with greater linkage of education and health data in Northern Ireland to understand the associations between these structures, whilst also ensuring a collaborative approach between such structures is implemented moving forward.

Education and health continue to be treated as separate issues in many governments, with limited collaboration or joint up responses that account for their interaction and impact on the outcomes and trajectories of students across their life course. Despite this argument being emphasised by Bloom [29], it remains of relevance in present day. The interactions between health and education must therefore be acknowledged by leaders in both areas to ensure a collaborative approach is implemented. This collaborative approach will ensure a unified response in the future which will have a short-term reciprocal effect on the current student cohort and a long-term impact on adults [30]. In Northern Ireland, there has been recent progress in this area. For example, the Children and Young People’s Emotional Health and Wellbeing in Education Framework [15] developed by the Department of Education and Department of Health provides guidelines to assist educational settings in promoting emotional wellbeing among students.

The self-report measure of health in this study highlights the influence of health status grading and the importance of establishing targeted interventions that are implemented early, are sustained and evidence informed. Such interventions can also be aligned with longitudinal evaluations to identify the most effective approaches for particular groups of students. In summary, across all four measures of health in the analysis, the need for targeted interventions to disrupt the downward educational trajectory of students with poorer physical health and mental health is clear. This research adds to the growing evidence that the relationship between poor mental health and educational attainment outcomes must be a key policy priority as it can impact upon individual’s later life outcomes in areas such as employment, income and housing [10].

## Conclusion

This novel study uses linked administrative data to examine how the physical and mental health of students can impact their educational attainment. This study is the first instance in which this relationship has been analysed using population wide data in the context of Northern Ireland. The results of the study highlight the importance of disaggregating not only the presence of, but also type of, physical health conditions and mental health conditions to gain a holistic understanding of how health can impact upon educational attainment. The study is dependent upon the health measures collected in the 2011 household Census. Although these measures provide high-level, generalised and somewhat blunt indicators of the health status of individuals, each health indicator included in this analysis reaffirms the associations between lower post-primary attainment and poorer health, which is evidenced in the international context. However, unique to this study is the inclusion of measures that account for an individual’s self-reported health status, the presence of a physical health condition, the presence of a mental health condition *and* the presence of a limiting illness. Of particular importance is the magnitude of the effect mental health has on educational outcomes. As noted, this is a pertinent area of policy attention and action, not only locally in Northern Ireland but globally. However, when considering policy, strategies, or programmes to target the interlinking health and education inequalities, it is important that consideration is provided to the potentially greater disadvantage some social groups experience, as reflected through the interactions of health conditions with sex and FSME.

## Supplementary Information

Supplementary Material 1.

## Data Availability

The data used for analysis in this study are not publicly available. The data were provided for the sole purpose of this study from the data suppliers.

## Abbreviations

FSM: Free School Meals
FSME: Free School Meal Eligibility
GCSE: General Certificate of Secondary Education
NI-MDM: Northern Ireland Multiple Deprivation Measure
UK: United Kingdom
WHO: World Health Organisation

## Footnotes

1 DAG was created using DAGitty (https://www.dagitty.net/).

2 The analysis did not account multiple testing but relies on both the statistical significance and the magnitude of the contribution of each variable in the model when discussing its effects.
