# Supplementary material for: Healthy lives, enriched minds: the role of physical health and mental health on educational attainment in Northern Ireland
Source: BMC Public Health. 2025 Aug 22;25:2886. doi: 10.1186/s12889-025-23780-3 (PMC12372220; doi:10.1186/s12889-025-23780-3)
Supplement: Supplementary file 1 — Supplementary Material 1. [file 12889_2025_23780_MOESM1_ESM.docx]

**Appendix**

The multilevel models are expressed through the following formula:

y_ij_ = b_0j_ + b_1_x_ij_ + b_2_x_j_ +u_j_ + e_ij_

where *yij* is the outcome measure of GCSE attainment for student *i* in school *j*, *b0j* is the intercept*,* *b1xij* is the individual-level predictor of attainment for student *i* in school *j,* *b2xj* is the school-level predictor of attainment*,* *uj* is the school-level residuals*, and eij* is the student-level residuals.
